# Development and Evaluation of Bioconverted Milk with Anti-Microbial Effect against Periodontal Pathogens and α-Glucosidase Inhibitory Activity

**DOI:** 10.3390/microorganisms12071290

**Published:** 2024-06-25

**Authors:** Yewon Lee, Yohan Yoon, Kyoung-Hee Choi

**Affiliations:** 1 Risk Analysis Research Center, Sookmyung Women’s University, Seoul 04310, Republic of Korea; yw0322@naver.com; 2 Department of Food and Nutrition, Sookmyung Women’s University, Seoul 04310, Republic of Korea; 3 Department of Oral Microbiology, College of Dentistry, Wonkwang University, Iksan 54538, Republic of Korea

**Keywords:** bioconverted milk, probiotics, *Artemisia herba-alba*, periodontal pathogens, α-glucosidase

## Abstract

To decrease periodontal pathogens and increase the number of beneficial bacteria, probiotics and bioactive compounds made via microbial bioconversion are recently used. In addition, the interest regarding probiotics-mediated bioconversion with popular medicinal plants is increasing. *Artemisia herba-alba*, a type of wormwood, has recently been attention as a medicinal plant due to its various bioactive compounds. Therefore, we developed bioconverted milk containing *A. herba-alba* that effectively inhibited periodontal pathogens and α-glucosidase. To select the appropriate lactic acid bacteria for the probiotic candidate strain, 74 strains of lactic acid bacteria were screened. Among them, *Lactiplantibacillus plantarum* SMFM2016-RK was chosen as the probiotic due to its beneficial characteristics such as high acid and bile tolerance, antioxidant activity, and α-glucosidase inhibition. Based on the minimal bactericidal concentration against three periodontal pathogens, the following appropriate concentrations of *Artemisia herba-alba* extract were added to milk: 5 mg/mL of *A. herba-alba* ethanol extract and 25 mg/mL of *A. herba-alba* hot-water extract. Four bioconverted milks (BM), BM1, BM2, BM3, and BM4, were produced by combining *L. plantarum* SMFM2016-RK alone, *L. plantarum* SMFM2016-RK and ethanol extract, *L. plantarum* SMFM2016-RK and hot-water extract, and *L. plantarum* SMFM2016-RK with both extracts. As a result of antimicrobial activity, BM3 inhibited the growth of *Aggregatibacter actinomycetemcomitans* and *Porphyromonas gingivalis* the most, and BM4 suppressed the growth of *Fusobacterium nucleatum* the most. In addition, bioconverted milk containing *A. herba-alba* (BM2, BM3, and BM4) inhibited α-glucosidase more effectively than BM1. The whole genome of *L. plantarum* SMFM2016-RK was obtained, and 3135 CDS, 67 tRNA, and 16 RNA were predicted. The genome annotation of *L. plantarum* SMFM2016-RK revealed 11 CDS related to proteolysis and amino acid metabolism and 2 CDS of phenolic acid-metabolizing enzymes. In conclusion, *A. herba-alba*-added milk bioconverted by *L. plantarum* SMFM2016-RK displayed both the growth inhibitory effect on periodontal pathogens and the α-glucosidase inhibitory activity; thus, it necessitates to evaluate the effects on the alleviation of periodontal diseases and glycemic control through future animal experiments.

## 1. Introduction

Oral disease is a major global health concern that affects a large portion of the world’s population. According to a report by the World Health Organization (WHO), almost half of the world’s population suffers from oral diseases [1]. Furthermore, oral infections and associated inflammatory responses can have a negative impact on blood glucose levels, and a previous study reported that more than 90% of diabetic patients experienced oral complications [2,3]. The α-glucosidase enzyme affects blood glucose levels in people with oral infections. It plays a major role in raising blood sugar, breaks down complex carbohydrates into simple sugars, and then absorbs them into the bloodstream. Therefore, it is known that inhibiting the α-glucosidase enzyme helps to prevent glucose levels from rising rapidly by allowing glucose to be gradually absorbed into the bloodstream [4]. Thus, many researchers have made an effort to identify potent α-glucosidase inhibitors [5,6,7,8]. The treatment and prevention of periodontitis involve various medications and therapeutic approaches. Some of the commonly used medications include antibiotics, antiseptics, and carbamide peroxide topical [9]. However, these treatments have certain limitations such as antibiotic resistance [9]. Due to the increase in antibiotic resistance, the focus of periodontitis treatment is on restoring the balance between the oral microbiota and the host’s periodontal tissue [9]. For this reason, probiotics markets targeting oral health are experiencing significant growth and development. The market size for probiotics specifically for oral health was valued at USD 2.5 billion in 2022 [10]. This growth is driven by research into specific probiotic strains, such as *Lactobacillus* and *Bifidobacterium* species, which have shown potential benefits in reducing the risk of dental caries, gum disease, and bad breath [11]. In the food industry, *Lactiplantibacillus plantarum* (formerly *Lactobacillus plantarum*) is one of the lactic acid bacteria (LAB) that is often used as a potential probiotic starter. As its main metabolites, lactic acid, succinic acid, and acetic acid are produced by this non-spore-forming bacterium [12]. According to certain research, using *L. plantarum* specifically was linked to better periodontal health, as seen by smaller pockets and less bleeding when the gums were probed [13,14]. In addition, *L. plantarum* could reduce oral carriage of *Streptococcus mutans* [15,16]. Furthermore, various new quality-related components such as phenolic acids, flavones and their glycosides, alkaloids, and terpenoids have been identified due to changes in chemical composition during microbial bioconversion with plants in recent years [17]. Microbial bioconversion showed a synergistic effect compared to simple application of LAB, such as increasing the fermentation ability due to soybean protein and enhancing the inflammatory bowel disease-alleviating effect by boosting the bioactivity of anthocyanin [18,19].

*Artemisia herba-alba*, commonly known as desert or white wormwood, has been recognized for its potential medicinal properties [20]. Studies on this plant have identified various beneficial compounds such as herbalbin, *cis*-chryanthenyl acetate, flavonoids (hispidulin and cirsilineol), monoterpenes, and sesquiterpene [20]. It is used in folk medicine for treating a range of diseases [20]. Because of a very low level of toxicity, the aerial portions of *A. herba-alba* are appropriate for various uses [20]. This plant has shown promise in pharmacological and toxicological properties, but further studies are needed to integrate it more effectively into the healthcare system [20]. Therefore, this study aimed to select LAB as a probiotic strain and develop bioconversion products with *A. herba-alba* by the selected LAB that alleviate periodontal disease by inhibiting the growth of periodontal pathogenic bacteria and enhance glycemic control by impeding the α-glucosidase activity.

## 2. Materials and Methods

### 2.1. Selection of Probiotic Candidate Strain

#### 2.1.1. Preparation of Lactic Acid Bacteria Isolates

Seventy-four LAB isolates obtained from 106 kimchi samples [21] were screened to identify a candidate probiotic strain. The isolates were cultured in 10 mL Lactobacilli MRS broth (Becton, Dickinson and Company, Franklin, NJ, USA) and incubated at 35 °C for 24 h. The cultures were centrifuged at 1912× *g* (S750–4B swing rotor, Combi 514R, Hanil Science Inc., Gimpo, Republic of Korea) and at 4 °C for 15 min and washed twice with 10 mL phosphate buffered saline (PBS; pH 7.4; 0.2 g KCl, 0.2 g KH_2_PO_4_, 8.0 g NaCl, and 1.5 g Na_2_HPO_4_·7H_2_O in 1 L distilled water).

#### 2.1.2. Hemolytic Analysis

Hemolytic activity was analyzed by streaking fresh cultures on Columbia agar (BioMérieux, Marcy l’Etoile, Lyon, France), containing 5% sheep blood (*w*/*v*), and incubating them at 35 °C for 48 h. Blood agar plates were examined for signs of α-hemolysis (green-hued zones around colonies), β-hemolysis (clear zones around colonies), and γ-hemolysis (no zone around colonies).

#### 2.1.3. Analysis of β-Glucosidase and β-Glucuronidase Activities

β-glucosidase and β-glucuronidase enzymatic activities were evaluated using the API ZYM test kit (BioMérieux) according to the manufacturer’s instructions. The LAB isolates were diluted until their OD_500_ reached 1.048; 65 μL of the aliquots was then inoculated into the API ZYM test kit wells and incubated at 35 °C for 4 h. The results were graded from 0 (no activity) to 5 (>40 nanomoles) by comparing color intensity. Results with grades > 2 were considered positive.

#### 2.1.4. Analysis of Acid and Bile Salt Tolerance

For acid tolerance, 500 μL of washed bacterial pellets was inoculated in 500 μL Lactobacilli MRS broth adjusted to pH 2.5 and then incubated at 35 °C. Bacterial cells were counted after 0 and 3 h of exposure to acidic conditions. Aliquots of 100 μL were serially diluted and spread onto Lactobacilli MRS agar plates (Becton, Dickinson and Company). The bacterial colony-forming units were counted after 24 h of incubation at 35 °C. The results of acid tolerance were compared to those of *Lacticaseibacillus rhamnosus* GG (LGG, ATCC53103). To evaluate bile salt tolerance, 500 μL culture was inoculated in 500 μL Lactobacilli MRS broth, containing 3% oxgall (Becton, Dickinson and Company), in 96-well microplates (SPL Life Sciences, Pocheon, Republic of Korea) and incubated at 35 °C. After 24 h of incubation, 100 μL of the aliquots were serially diluted and plated on Lactobacilli MRS agar. The bacterial cells were counted after 24 h of incubation at 35 °C. These results were compared to those of LGG.

#### 2.1.5. Determination of ABTS-Scavenging Activity

The mixture of 2.6. mM potassium persulfate and 7.4 mM 2,2′-azino-bis (3-ethylbenzothiazoline-6-sulfonic acid) (ABTS; Sigma-Aldrich, St. Louis, MO, USA) solutions was prepared in a 1:1 ratio. The mixture was placed in the dark for 20 h until it became blue-green owing to radical formation. The mixture was diluted until its OD_734nm_ reached 0.7 ± 0.02. Approximately 500 μL of the isolates (9 log CFU/mL) was added to the solution (500 μL) and incubated in the dark at 37 °C for 30 min. The absorbance of the samples was measured at 734 nm using a microplate spectrophotometer (BioTek Instruments Inc., Winooski, VT, USA). The ABTS-scavenging activity of the isolates was calculated as follows; ABTS radical scavenging (%) = {(OD_734nm_ of control − OD_734nm_ of sample)/OD_734nm_ of control} × 100.

### 2.2. Preparation of Artemisia herba-alba Extracts

Hot water and ethanol were used to extract the hydrophilic and hydrophobic compounds from the dried *A. herba-alba* leaves (Bedel Food, Incheon, Republic of Korea), respectively. The hydrophobic compounds were extracted from 100 g of dried *A. herba-alba* in 1 L of 95% ethanol (Samchun, Pyeongtaek, Republic of Korea) at 60 °C for 24 h. The hydrophilic compounds were extracted from 100 g of dried *A. herba-alba* in 1 L of distilled water at 90 °C for 1 h. The extracts were filtered with Advantec NO. 1 filter paper (Advantec Toyo Kaisha Ltd., Taito-ku, Tokyo, Japan), and the filtrates were concentrated through rotary evaporation (Laborota 4001 WB, Vertrieb, Germany) under vacuum at 80 °C. The concentrates were frozen at −80℃ for 24 h, lyophilized at −80 °C for 3 days using a freeze dryer (FDB-5503, Operon Co Ltd., Gimpo, Republic of Korea), and the powder was collected.

#### 2.2.1. Minimum Bactericidal Concentration of *Artemisia herba-alba* Extracts against Periodontal Pathogens

To select the optimal concentrations of the two *A. herba-alba* extracts, the minimum bactericidal concentration (MBC) was analyzed. The standard broth dilution method (CLSI M07-A8) was used to determine the antimicrobial efficacy of *A. herba-alba* extracts by evaluating the growth of the following periodontal microbial pathogens: *Aggregatibacter actinomycetemcomitans* ATCC43718, *Fusobacterium nucleatum* ATCC10953, and *Porphyromonas gingivalis* ATCC33277. The strains were cultured in 10 mL Wilkins Chalgren Anaerobe Broth (Oxoid, Basingstoke, Hampshire, UK) and incubated at 35 °C for 48 h in a 90% N_2_, 5% CO_2_, and 5% H_2_ atmosphere. The cells were collected through centrifugation at 1912× *g* and 4 °C for 15 min and washed twice with 10 mL PBS. The two types of *A. herba-alba* extracts were diluted in Brain Heart Infusion broth (Becton, Dickinson and Company) to concentrations of 102.4–0.4 mg/mL and dispensed into a 96-well microplate. Periodontal bacterial cultures were inoculated into all wells, and the plates were incubated as described above. The culture was streaked on Columbia agar containing 5% sheep blood (*w*/*v*) and incubated as described above. The MBC was determined based on colony formation.

#### 2.2.2. Growth of Probiotic Candidate Strains in the Presence of *Artemisia herba-alba* Extracts

To determine whether *A. herba-alba* extracts inhibit the growth of LAB, the growing ability of the isolates when cocultured with *A. herba-alba* was evaluated. The LAB isolates, including *Lactilactobacillus curvatus* SMFM2016-NK, which was previously identified as an effective anti-periodontitis isolate [22], were diluted to 3.0 ± 0.5 Log CFU/mL using buffered peptone water (Becton, Dickinson and Company). The diluted aliquots were inoculated into four different liquid media—Lactobacilli MRS broth, Lactobacilli MRS broth containing 25 mg/mL *A. herba-alba* ethanol extract, Lactobacilli MRS broth containing 5 mg/mL *A. herba-alba* hot-water extract, and Lactobacilli MRS broth containing 25 mg/mL *A. herba-alba* ethanol extract and 5 mg/mL *A. herba-alba* hot-water extract. The samples were incubated at 37 °C for 20 h, and they were plated on Lactobacilli MRS agar. The bacterial cells were enumerated after a 24 h incubation at 35 °C. The viable cell counts were used to calculate the growth rate using the following formula:

Growth rate = {LN(D_2_) − LN(D_1_)}/(t_2_ − t_1_)

where t_1_ denotes 0 h; t_2_ denotes 20 h; D_1_ represents the number of viable cells at 0 h; and D_2_ represents the number of viable cells at 20 h.

The mean generation time (Td) was calculated as Td = LN(2)/growth rate [23].

#### 2.2.3. Antimicrobial Effects of *Artemisia herba-alba* Cocultured Broths against Periodontal Pathogens

Coculture broths containing *A. herba-alba* extracts and LAB were analyzed for their antimicrobial effects against periodontal pathogens. Three types of broth (10 mL) were prepared—Lactobacilli MRS broth, Lactobacilli MRS broth containing 5 mg/mL *A. herba-alba* ethanol extract, and Lactobacilli MRS broth containing 25 mg/mL *A. herba-alba* hot-water extract. These broths, along with the probiotic candidate strains, were cultured at 35 °C for 24 h. *A. actinomycetemcomitans* ATCC43718 and *F. nucleatum* ATCC10953 were inoculated into 10 mL Wilkins Chalgren Anaerobe Broth (Oxoid) and incubated at 35 °C for 48 h in a 90% N_2_, 5% CO_2_, and 5% H_2_ atmosphere. The cell pellets of periodontal bacteria were harvested through centrifugation at 1912× *g* at 4 °C for 15 min and washed twice with 10 mL PBS. One hundred microliters of the aliquots were plated on Columbia agar (BioMérieux) and dried for 30 min; then, 10 µL bioconversion broth was spot-inoculated on Columbia agar and incubated at 35 °C for 48 h in a 90% N_2_, 5% CO_2_, and 5% H_2_ atmosphere. The inhibition zone from the edge of the spot to the clear zone was measured.

#### 2.2.4. α-Glucosidase Inhibitory Activity of *Artemisia herba-alba* Cocultured Broths

To analyze the α-glucosidase inhibitory activity, pre-reaction mixtures comprising 50 μL coculture broth samples, 50 μL of 200 mM phosphate buffer (pH 6.5; Sigma-Aldrich), and 50 μL of 0.75 units/mL α-glucosidase (Sigma-Aldrich) were prepared. The reaction mixture was incubated at 37 °C for 10 min. Subsequently, the reaction was initiated by adding 100 μL p-nitrophenyl α-glucopyranoside (Sigma-Aldrich) diluted 10 times in dimethyl sulfoxide (Duksan Pure Chemicals Co., Ltd., Ansan, Republic of Korea), followed by incubation at 37 °C for 10 min. The reaction was terminated by adding 750 μL of 0.1 M sodium carbonate (Duksan Pure Chemicals Co., Ltd.). p-nitrophenyl release was assessed by measuring the absorbance at 405 nm (BioTek Instruments Inc.). A control sample was prepared by replacing the coculture sample with sterile distilled water. Percentage of α-glucosidase inhibition was calculated using the following formula:

α-glucosidase inhibitory activity (%) = {1 − (OD_405nm_ of sample/OD_405nm_ of control)} × 100.

### 2.3. Preparation of Bioconverted Milk

Four bioconverted milk (BM) samples, including milk with 9 Log CFU/mL of *L. plantarum* SMFM2016-RK (BM1), BM1 and 5 mg/mL *A. herba-alba* ethanol extract (BM2), BM1 and 25 mg/mL *A. herba-alba* hot-water extract (BM3), and BM1, 5 mg/mL *A. herba-alba* ethanol extract, and 25 mg/mL *A. herba-alba* hot-water extract (BM4), were prepared as follows. Approximately 100 mL of 10% skim milk (Becton, Dickinson and Company) containing 0.5% yeast extract (Becton, Dickinson and Company) was pasteurized at 100 °C for 10 min. The pasteurized milk was cooled to 40 °C. Then, 5 mg/mL *A. herba-alba* ethanol extract, 25 mg/mL *A. herba-alba* hot-water extract, and both 5 mg/mL *A. herba-alba* ethanol extract and 25 mg/mL *A. herba-alba* hot-water extract were added in the milk to prepare BM2, BM3, and BM4, respectively. The milk was stirred for 1 min. Then, 5 mL of the *L. plantarum* SMFM2016-RK inoculum was inoculated, and the milk was stirred again for 1 min. The inoculated milk samples were incubated at 42 °C; when the pH of the BM fell below 5.0, the samples were stored at 4 °C for 24 h. The BMs were stored at −80 °C for 24 h, and whole frozen BMs were lyophilized at −80 °C for 3 days. The lyophilized BM samples were dissolved in sterile distilled water at a concentration of 1 g/10 mL for further use.

#### 2.3.1. Antimicrobial Effects of Bioconverted Milk against Periodontal Pathogens

The antimicrobial effects of four types of BM samples against periodontal pathogens were analyzed using the paper disc diffusion inhibition method. *A. actinomycetemcomitans* ATCC43718, *F. nucleatum* ATCC10953, and *P. gingivalis* ATCC33277 were prepared as described in Section 2.2.1. Approximately 100 μL of the aliquots was plated on Columbia agar and air-dried for 30 min. Sterilized paper discs were placed at different areas on the surface of the plate. Then, 10 µL BMs were spot-inoculated on the paper disc and dried for 10 min. The dried plates were incubated at 35 °C for 48 h in a 90% N_2_, 5% CO_2_, and 5% H_2_ atmosphere. The inhibition zone, from the edge of the disc to the clear zone, was measured.

#### 2.3.2. Analysis of the α-Glucosidase Inhibitory Activity of Bioconverted Milk

The α-glucosidase inhibitory activity of the four types of BM samples was analyzed as described in Section 2.2.4.

### 2.4. Whole-Genome Analysis of Novel Probiotics

#### 2.4.1. DNA Extraction and Library Preparation

DNA of the selected LAB was extracted with the DNeasy Blood and Tissue Kit (Qiagen, Hilden, Germany) in accordance with the manufacturer’s instructions. Library was constructed with 5 μg of the extracted DNA sample using the SMRTbell™ Template Prep Kit 1.0 (PN 100-259-100) (Pacific Biosciences, Menlo Park, CA, USA) in accordance with the manufacturer’s instructions. Using the BluePippin size selection technique (Sage Science, Beverly, MA, USA), fragments of the SMRTbell template less than 20 kb were eliminated to create large-insert libraries. For quality control, the generated library was confirmed using an Agilent 2100 Bioanalyzer (Santa Clara, CA, USA).

#### 2.4.2. De Novo Sequencing

The DNA/Polymerase Binding Kit P6 (Pacific Biosciences) was used to bind DNA polymerase to the complex after the SMRTbell libraries were annealed to sequencing primers. The PacBio RS II sequencing platform (Pacific Biosciences) was used to sequence the polymerase-SMRT bell adaptor complex after it was inserted into SMRT cells. De novo assembly was used to create long contigs, and then gene annotation and prediction were carried out to examine their genetic characteristics.

#### 2.4.3. Comparison with Other Lactic Acid Bacteria

The sequence of the final probiotic isolate, identified as *L. plantarum*, was compared with those of seven other *L. plantarum* strains (E1, MF1298, SRCM103473, SRCM103472, K259, NCU116, and CNEI-KCA4) that were shown to have high similarity through BLAST analysis by the NCBI GenBank database. Chromosomal characteristics, such as isolated source and location, chromosomal genome size, and the number of tRNAs and rRNAs, of *L. plantarum* were compared among the strains. The sequence data of seven *L. plantarum* strains were downloaded from the NCBI to calculate the average nucleotide identity (ANI, %) among the chromosomal DNA of the *L. plantarum* strains, using the unweighted pair group method with arithmetic mean tree, created using the CLC program (Insilicogen, Yongin, Republic of Korea).

## 3. Results and Discussion

### 3.1. Determination of A. herba-alba Extract Concentration

*A. herba-alba* ethanol extract exhibited the highest antimicrobial effect against the periodontal pathogen *P. gingivalis*, with an MBC of 1.4 mg/mL. Its MBCs against *F. nucleatum* and *A. actinomycetemcomitans* were 4.3 and 5.3 mg/mL, respectively (Table 1). *A. herba-alba* hot-water extract exhibited the highest antimicrobial effect against *F. nucleatum*, with an MBC of 5.9 mg/mL. Its MBCs against *P. gingivalis* and *A. actinomycetemcomitans* were 26.5 and 10.7 mg/mL, respectively. There was a difference between ethanol extract and hot-water extract as a result of the MBC test of *A. herba-alba* extracts. *A. herba-alba* contains various bioactive compounds, including phenolic compounds [24], and there were shown different minimum inhibitory concentrations of compounds from different fractions for *Bacillus cereus*, *Escherichia coli*, *Staphylococcus aureus*, and *Proteus vulgaris* [25]. Thus, depending on the extraction solvent, different components might have been extracted, resulting in varying the antimicrobial activity in this study. Another cause for the difference in antibacterial activity across extracts could be whether a concentration technique was used to produce the extract. The *A. herba-alba* ethanol extract was concentrated to remove the solvent after extraction and then lyophilized, whereas the hot-water extract was directly lyophilized. Another possible cause may be the difference between compositions of antimicrobial substances contained in two extracts. The bioactive components extracted from plants generally vary based on the extraction solvent and method. The extraction yields also vary; therefore, desirable antimicrobial activities appear at different concentrations [26]. Accordingly, 5 mg/mL *A. herba-alba* ethanol extract and 25 mg/mL *A. herba-alba* hot-water extract efficiently were used to prepare bioconversion products with the chosen probiotic candidate strain.

### 3.2. Selection of Probiotic Candidate Strain

**Figure 1:**
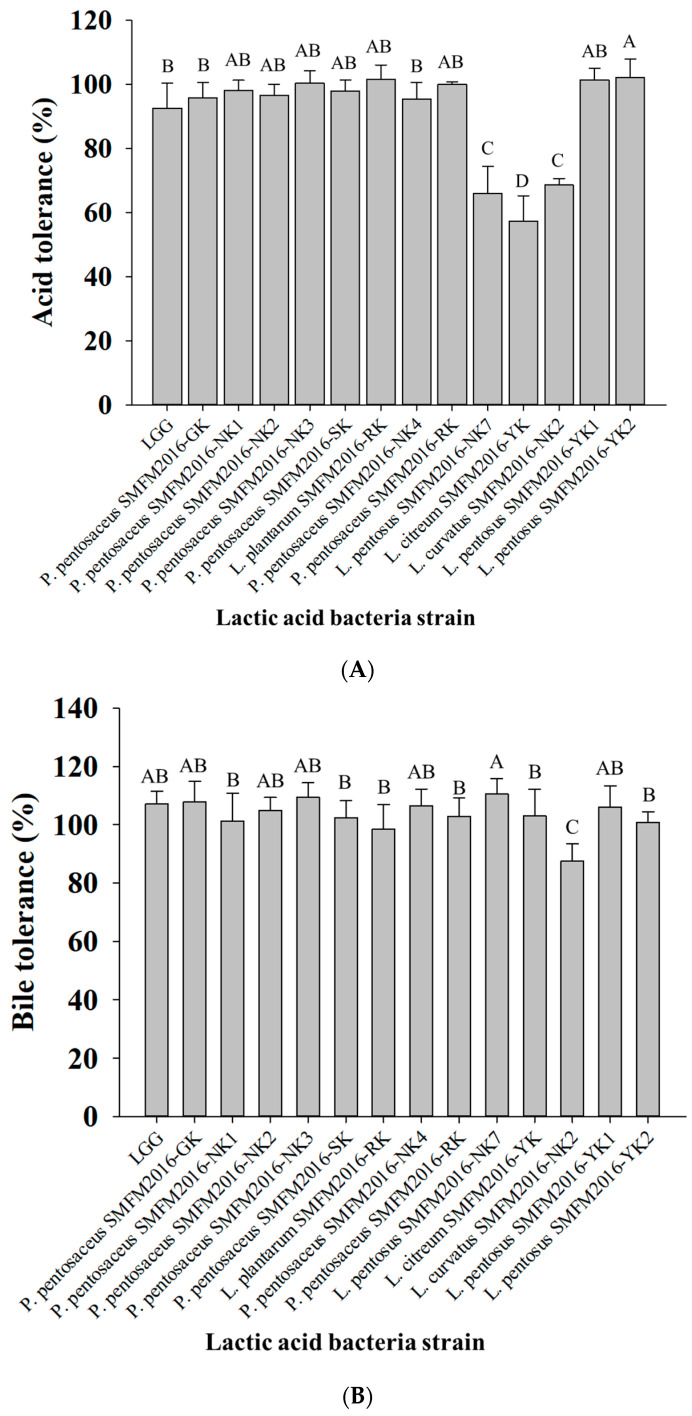
Acid tolerance (**A**) and bile salt tolerance (**B**) of the lactic acid bacteria isolates. A–D; different letters indicate a significant difference (*p* < 0.05).

α-hemolysis and β-hemolysis are major virulence indicators of pathogenic bacteria, while LAB isolates with γ-hemolytic activity are considered safe for consumption, as they exhibit low virulence [27]. Hemolytic analysis is essential to determine whether a strain can be safely used by humans and animals as probiotics [28]. Among the 74 LAB isolates examined in this study, 35 isolates exhibited γ-hemolysis, 36 isolates exhibited α-hemolysis, and 3 isolates exhibited β-hemolysis. The 35 isolates showing γ-hemolysis were selected for further analyses of β-glucuronidase and β-glucosidase activities. Thirteen strains were negative to produce β-glucosidase and β-glucuronidase and were selected as candidate probiotic strains. When the toxic substances enter the body, β-glucoside and glucuronic acid molecules are transferred from the liver to the colon as glucuronic acid conjugates after being detoxified [29]. However, in the colon, β-glucuronidase disrupted this connection, producing amines, poisons, or mutations that could act as carcinogens [29]. β-glucosidase hydrolyzes glycosides, and the undigested glycosides are transported to the colon, where bacterial β-glucosidase further hydrolyzes them [30]. Aglycones formed during this conversion are often toxic and carcinogenic [30]. Therefore, both are considered harmful enzymes [31]. The 13 isolates were assessed for their tolerance to acid and bile salt. The acid and bile salt tolerance of the 13 isolates was 56.6–102.2% and 87.5–110.5%, respectively (Figure 1). The acid tolerance of most isolates, except that of *Lactobacillus pentosus* SMFM2016-NK7 (66.0%), *Leuconostoc citreum* SMFM2016-YK (57.3%), and *L. curvatus* SMFM2016-NK2 (68.6%), was higher than that of LGG (92.6%) (Figure 1A). In the bile tolerance analysis, *L. pentosus* SMFM2016-NK7 showed the highest tolerance of 110.5%, which was significantly higher (*p* < 0.05) than that of LGG (107.1%) (Figure 1B). Additionally, all isolates, except *L. curvatus* SMFM2016-NK2 (87.5%), did not show a significant difference when compared to LGG. Therefore, the *L. pentosus* SMFM2016-NK7, *L. citreum* SMFM2016-YK, and *L. curvatus* SMFM2016-NK2 isolates, which showed significantly lower (*p* < 0.05) tolerance than LGG, were excluded.

**Figure 2:**
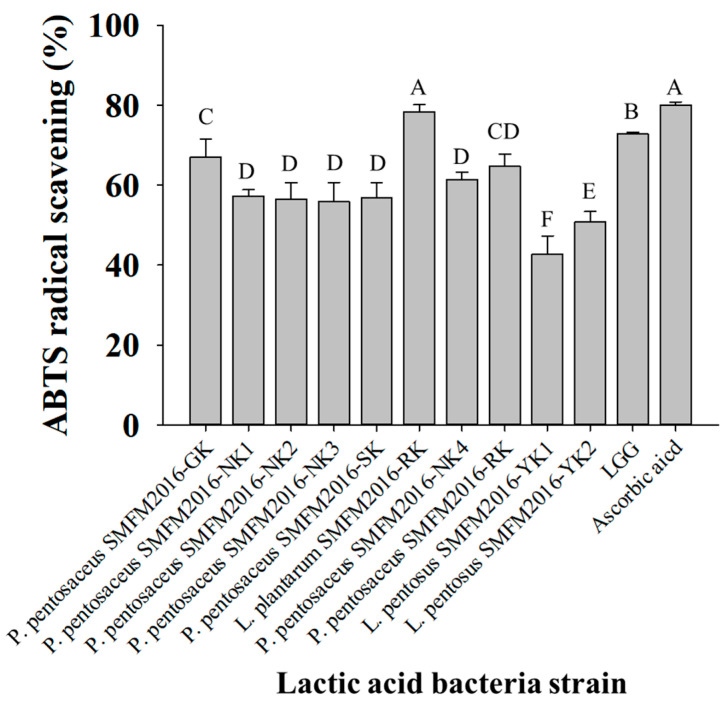
2,2′-azinobis (3-ethylbenzothiazoline-6-sulfonic acid; ABTS) scavenging activity of the lactic acid bacteria isolates. A–F; different letters indicate a significant difference (*p* < 0.05).

The remaining ten isolates were analyzed for ABTS radical-scavenging activity; their activities were higher than 40% (Figure 2). Among the ten isolates, *L. plantarum* SMFM2016-RK showed the highest activity (78.4%), with no significant difference in 0.2 mM ascorbic acid (79.8%). *L. pentosus* SMFM2016-YK1 (42.7%) and *L. pentosus* SMFM2016-YK2 (50.8%) exhibited significantly lower (*p* < 0.05) activities than the other bacteria. Therefore, these two strains were excluded from the list of probiotic candidate strains in this study, and the remaining eight isolates were subjected to further analysis.

Among the nine LAB isolates, including *L. curvatus* SMFM2016-NK, the growth rate averaged 0.051 and the mean generation time was 13.5 h (Table 2 and Table 3). None of the isolates showed significantly altered growth rates upon addition of the two types of *A. herba-alba* extracts. However, the growth rate of LGG in *A. herba-alba* ethanol extract was significantly lower, and its generation time in *A. herba-alba* ethanol extracts was significantly longer (*p* < 0.05) than that in the MRS broth or *A. herba-alba* hot-water extract. This indicated that *A. herba-alba* ethanol extract suppressed the growth of LGG.

The antimicrobial analysis of the ethanol or hot-water extract against the nine isolates revealed that the extracts bioconverted by *L. plantarum* SMFM2016-RK or *L. curvatus* SMFM2016-NK exhibited a higher inhibitory effect *on A. actinomycetemcomitans* ATCC43718 and *F. nucleatum* ATCC10953 than those bioconverted by other isolates (Table 4). *L. plantarum* SMFM2016-RK and *L. curvatus* SMFM2016-NK were more effective on inhibiting the growth of two periodontal pathogens in a coculture broth with *A. herba-alba* extracts when compared to the isolates grown in MRS broth. Among the cultures, the culture broth of *L. plantarum* SMFM2016-RK with *A. herba-alba* ethanol extract showed the highest inhibitory effect against *A. actinomycetemcomitans* ATCC43718, with an inhibition zone of 2.1 mm (*p* < 0.05). The culture broth of *L. curvatus* SMFM2016-NK with *A. herba-alba* ethanol extract exhibited the highest inhibition size of 4.0 mm against *F. nucleatum* ATCC10953 (*p* < 0.05). Although it was difficult to evaluate the antimicrobial effect of LAB and *A. herba-alba* extracts against *P. gingivalis*, their effects on the inhibition of *P. gingivalis* biofilm were observed. Probiotic-mediated bioconversion releases bioactive metabolites, such as antimicrobial peptides, immunopeptides, and bioactive polyphenols, that are effective on maintaining oral health [32,33,34]. Therefore, *A. herba-alba* coculture broths containing *L. plantarum* SMFM2016-RK or *L. curvatus* SMFM2016-NK could be considered for a possible alleviator of periodontal disease, as they inhibit the growth of periodontal pathogens. Hence, *L. plantarum* SMFM2016-RK and *L. curvatus* SMFM2016-NK were selected for analyzing α-glucosidase activity. α-glucosidase inhibition disrupts carbohydrate digestion and absorption, thereby alleviating hyperglycemia, and reduces the angiotensin-converting enzyme inhibitory potential [35,36]. Accordingly, the isolate with a high α-glucosidase activity could be effective in alleviating the systemic disease caused by periodontitis. The α-glucosidase inhibitory effects vary from 0 to 85.23% for the different strains of LAB [36,37]. *L. plantarum* SMFM2016-RK cultured in MRS broth displayed the highest α-glucosidase inhibitory activity of 85.2 ± 0.3% (Table 5). The three coculture broths containing *L. curvatus* SMFM2016-NK showed significantly lower activities than the other coculture broths (*p* < 0.05). Since this study examined the α-glucosidase inhibitory activity at a constant concentration of *A. herba-alba*, it is difficult to compare its activity with that in other studies that showed the concentration of IC_50_. According to Dar et al. (2024), α-glucosidase inhibitory activity increased with *Capsella bursa-pastoris* ethanol extract [38]. Thus, it is expected that the inhibitory effect would increase in association with the *A. herba-alba* concentration. Eventually, *L. plantarum* SMFM2016-RK was chosen as the probiotic candidate strain to execute the bioconversion of *A. herba-alba*. Both types of *A. herba-alba* extracts were effective on different functionalities; therefore, both extracts were used for developing BM.

### 3.3. Efficacy Evaluation of Bioconverted Milk

When the BM reached an optimum pH of 4.5–5.0, it was considered fermented. The pH of milk containing *A. herba-alba* extracts and *L. plantarum* SMFM2016-RK (BM2, BM3, and BM4) was slightly lower than that of milk without addition of *A. herba-alba* extracts (BM1) at 0 h (Table 6). BM3 and BM4 reached the optimum pH of 4.62 ± 0.30 and 4.58 ± 0.25, respectively, after 6 h of fermentation, and the LAB cell count was 9.2 ± 0.1 Log CFU/mL. Collectively, *A. herba-alba* hot-water extract exhibited a synergistic effect that improved the growing ability of *L. plantarum* SMFM2016-RK. BM1 reached a pH of 4.42 ± 0.57 after 24 h, and the LAB cell count was 8.8 ± 0.2 Log CFU/mL. BM2 reached a pH of 4.58 ± 0.50 only after a long fermentation time of 37 h, and the LAB cell count was 8.8 ± 0.3 Log CFU/mL. Therefore, different fermentation conditions were applied to produce each type of BM. The prepared BM samples were analyzed for their antimicrobial effects against periodontal pathogens.

The inhibition zones of *A. actinomycetemcomitans* ATCC43718, *F. nucleatum* ATCC10953, and *P. gingivalis* ATCC33277 were 2.6−3.6, 1.6−2.9, and 1.6−1.9 mm, respectively (Table 7). The average inhibition zones for periodontal pathogens when using BM1, BM2, BM3, and BM4 were 2.0 ± 0.8, 2.4 ± 0.8, 2.5 ± 1.1, and 2.6 ± 1.1 mm, respectively. Bioconversion products with *A. herba-alba* extracts (BM2, BM3, and BM4) exhibited marginally higher antimicrobial effects than those without the extract (BM1). Among these products, BM3 showed the highest antimicrobial activity against *A. actinomycetemcomitans*, with an inhibition zone of 3.6 ± 0.8 mm (*p* < 0.05). BM4 showed the highest inhibition zone of 2.5 ± 0.6 mm for *F. nucleatum* ATCC10953, which was significantly higher than that achieved with BM1 (1.6 ± 0.5 mm; *p* < 0.0.5). BM1 and BM3 showed the same inhibition zone of 1.9 ± 0.3 mm for *P. gingivalis* ATCC33277.

**Figure 3:**
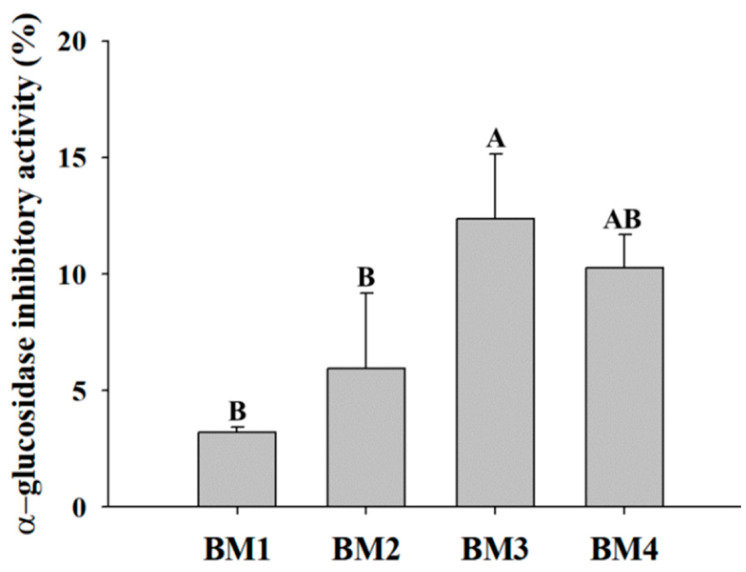
Comparison of α-glucosidase inhibitory activity on treatment with bioconverted milk. BM1: bioconverted milk with *L. plantarum* SMFM2016-RK, BM2: BM1 and 5 mg/mL *A. herba-alba* ethanol extract, BM3: BM1 and 25 mg/mL *A. herba-alba* hot-water extract, BM4: BM1, 5 mg/mL *A. herba-alba* ethanol extract, and 25 mg/mL *A. herba-alba* hot-water extract. A, B; different letters indicate a significant difference (*p* < 0.05).

Bioconversion products containing *A. herba-alba* extracts (BM2, BM3, and BM4) exhibited significantly higher *(p* < 0.05) α-glucosidase inhibitory activity than BM1 (Figure 3). Notably, BM3 displayed a significantly higher activity (12.4 ± 0.2%) than the other samples (*p <* 0.05). This study found that the bioconversion process of *A. herba-alba* hot-water extract and *L. plantarum* SMFM2016-RK enhanced the fermentation ability of LAB and inhibition activity against α-glucosidase and periodontal pathogens that could be the candidate material to alleviate periodontitis and glycemic control.

### 3.4. Whole-Genome Analysis of Novel Probiotics

**Figure 4:**
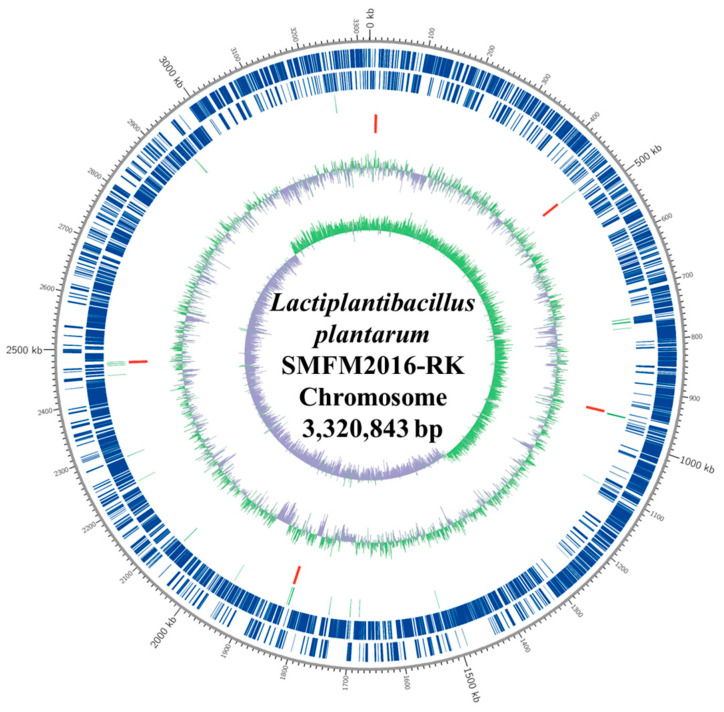
Chromosomal genome properties of *Lactiplantibacillus plantarum* SMFM2016-RK. Outer scale; base pairs, the first (the outer-most) and second ring; forward and reverse open reading frame (ORF), the third and fourth ring; forward and reverse ORF by gene annotation, the fifth and sixth ring; rRNA and tRNA genes, the seventh and eighth ring; positive and negative GC content values, the inner-most circle; GC skew.

*L. plantarum* SMFM2016-RK was sequenced using *de novo* assembly, and the whole genome was obtained. Three contigs—Contig1, Contig2, and Contig3—were assembled, and their lengths were 3,320,843 bp, 60,102 bp, and 39,997 bp, respectively. Using BLAST, the best match for *L. plantarum* SMFM2016-RK was identified as *Lactiplantibacillus plantarum*, with a 99.96% identification rate. For gene function analysis, gene annotation and gene prediction were performed using the information provided by the gene ontology database. Contig1 (chromosome) was predicted to contain 3135 coding sequences (CDS). The 67 tRNA and 16 rRNA genes and their locations were predicted from the CDS. The structural and functional properties of Contig1 were described using DNA plots (Figure 4). The predicted functional genes were divided into three primary categories—biological process, molecular function, and cellular component—according to their characteristics. Two thousand nine hundred and twelve transcripts were included under biological process; 2280 transcripts under molecular function; and 1378 transcripts under cellular component (Table 8). In the biological process, metabolic process had the highest number (1089) of transcripts. This included transcripts corresponding to various chemical reactions and pathways of organisms and the processes such as protein synthesis and degradation.

Gene annotation analysis using the evolutionary genealogy of genes: The non-supervised Orthologous Groups (EggNOG) database revealed that *L. plantarum* SMFM2016-RK had a high ability for carbohydrate transportation and metabolism (9.25%) and amino acid transportation and metabolism (6.57%; Table 9). *L. plantarum* SMFM2016-RK had some CDS related to proteolysis and amino acid metabolism (Table 10). The genes known as oligopeptide ABC transportation system (*oppF*, *oppD*, *dtpT*, *pepF1*, and *pepO*) and the serine-related metabolism (*glyA*, *dsdA*, and *sdhA*) were mostly identified. In addition, the arginosuccinate metabolism operon clustered with aspartate aminotransferase (*asp*), lyase (*argH*), and synthase (*argG*), which can be used for energy production and NADH regeneration, was also observed. These genes might influence various proteins and amino acids present in BM3, thus improving the growth of *L. plantarum* SMFM2016-RK and reducing the fermentation time. *L. plantarum* SMFM2016-RK was also shown to possess *lpdC* (gallate decarboxylase) and *padC* (phenolic acid decarboxylase), which encode phenolic acid-metabolizing enzymes (Table 11). In relation to this, several studies reported that phenolic acids were the main bioactive ingredients of *A. herba-alba* [25,39]. The phenolic compounds produced by LAB from precursors improve efficacy in the body or enable the production of bioactive metabolites through changes in gut microbiome [40,41]. According to Sun and Miao (2020), phenolic compounds can lower the glycemic index by altering the digestibility of food, and flavonoids and proanthocyanidins are effective in inhibiting α-glucosidase activity [42]. Likewise, in this study, it can be assumed that the inhibition of α-glucosidase by BM3 is due to the action of these phenolic metabolites produced during bioconversion of milk and *A. herba-alba* extracts by LAB.

**Figure 5:**
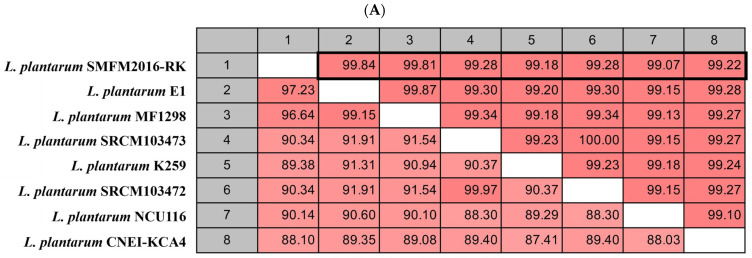

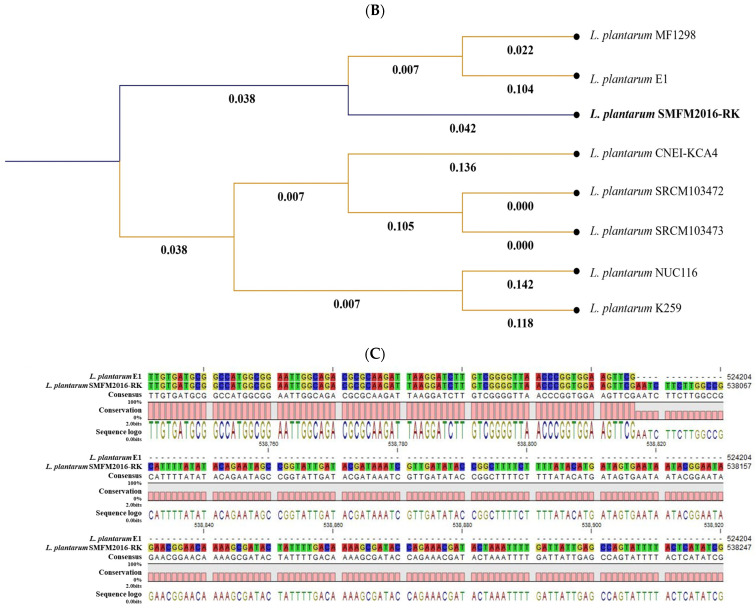
Average nucleotide identity (ANI) (**A**), unweighted pair group method with arithmetic mean (UPGMA) tree by average nucleotide identity (ANI) (**B**), and comparison of genome annotation with *Lactiplantibacillus plantarum* E1 (region 538,067-538,247) (**C**) of *L. plantarum* SMFM2016-RK.

The microbial genome similarity and the molecular phylogenetic relations were determined by analyzing the phylogenetic tree and ANI (%). The ANI of the seven *L. plantarum* strains in the NCBI GenBank database were compared to that of *L. plantarum* SMFM2016-RK (Figure 5A). The seven *L. plantarum* strains were isolated from various foods, and differences in genome size, GC content, and the numbers of tRNA and rRNA were observed. The ANI (%) between *L. plantarum* strains was 99.07–99.84%, and *L. plantarum* SMFM2016-RK was closest to the *L. plantarum* E1 strain, according to the unweighted pair group method with arithmetic mean tree by ANI (Figure 5A,B). Since ANI ≥ 96.5% is common in genomes within the same species, it was necessary to confirm whether *L. plantarum* SMFM2016-RK was a new strain [43]. Accordingly, whole genome annotation was performed with *L. plantarum* E1, which showed the highest similarity, using CLC workbench. Differences were observed between the two strains in various regions of the whole genome (Figure 5C). Therefore, *L. plantarum* SMFM2016-RK was confirmed to be a new strain.

## 4. Conclusions

This study identified *L. plantarum* SMFM2016-RK, which might be safe and inhibited the growth of periodontal pathogens and the activity of α-glucosidase. Whole-genome sequence analysis indicated that *L. plantarum* SMFM2016-RK is a new strain that could be used as a novel probiotic. The *A. herba-alba* extract added to skim milk was bioconverted by the newly characterized probiotic, and BMs containing *A. herba-alba* extracts (BM2, BM3, and BM4) showed the effects on impeding the growth of periodontal pathogenic bacteria and the activity of α-glucosidase. Taken together, probiotic-mediated bioconversion of *A. herba-alba* extract might be effective on periodontal disease and glycemic control. However, since the efficacy of the BM developed in this study was examined *in vitro*, it is necessary to apply it to animals in the future study to evaluate the efficacy *in vivo*.

## Figures and Tables

**Table 1:** Minimum bactericidal concentrations (mg/mL; mean ± standard deviation) of *Artemisia herba-alba* extracts against periodontal pathogens.

*Artermisia herba-alba* Extracts	*Fusobacterium nucleatum* ATCC43718	*Aggregatibacter actinomycetemcomitans* ATCC10953	*Porphyromonas gingivalis* ATCC33277	Total Average
Ethanol extracts	4.3 ± 1.7 ^C^	5.3 ± 1.7 ^C^	1.4 ± 0.4 ^C^	4.0 ± 2.1
Hot-water extracts	5.9 ± 1.3 ^C^	10.7 ± 3.3 ^B^	26.5 ± 2.0 ^A^	13.6 ± 9.1

^A–C^; different letters indicate a significant difference (*p* < 0.05).

**Table 2:** Specific growth rate of lactic acid bacteria in MRS broth containing different *Artemisia herba-alba* extracts (mean ± standard deviation).

Lactic Acid Bacteria Strain	MRS Broth	MRS Broth + *A. herba-alba* Ethanol Extract (5 mg/mL)	MRS Broth + *A. herba-alba* Hot-Water Extract (25 mg/mL)
*Pediococcus pentosaceus* SMFM2016-GK	0.054 ± 0.002 ^Aa^*	0.052 ± 0.002 ^Aab^	0.052 ± 0.002 ^Aab^
*P. pentosaceus* SMFM2016-NK1	0.052 ± 0.002 ^Aab^	0.051 ± 0.003 ^Aab^	0.050 ± 0.001 ^Aab^
*P. pentosaceus* SMFM2016-NK2	0.052 ± 0.001 ^Aab^	0.052 ± 0.004 ^Aab^	0.052 ± 0.003 ^Aab^
*P. pentosaceus* SMFM2016-NK3	0.053 ± 0.002 ^Aab^	0.051 ± 0.002 ^Aab^	0.051 ± 0.002 ^Aab^
*P. pentosaceus* SMFM2016-SK	0.053 ± 0.001 ^Aa^	0.053 ± 0.004 ^Aab^	0.052 ± 0.002 ^Aab^
*Lactiplantibacillus plantarum* SMFM2016-RK	0.049 ± 0.001 ^Ab^	0.048 ± 0.002 ^Ab^	0.049 ± 0.002 ^Ab^
*P. pentosaceus* SMFM2016-NK4	0.053 ± 0.002 ^Aa^	0.052 ± 0.002 ^Aab^	0.052 ± 0.002 ^Aab^
*P. pentosaceus* SMFM2016-RK	0.053 ± 0.002 ^Aab^	0.050 ± 0.002 ^Aab^	0.050 ± 0.002 ^Aab^
*Lactilactobacillus curvatus* SMFM2016-NK	0.050 ± 0.002 ^Ab^	0.050 ± 0.001 ^Ab^	0.049 ± 0.002 ^Ab^
*Lacticaseibacillus rhamnosus* GG	0.042 ± 0.004 ^Ac^	0.028 ± 0.006 ^Bc^	0.44 ± 0.004 ^Ac^

* Means with the same row with different superscript capital letters and same column with different superscript small letters are significantly different (*p* < 0.05).

**Table 3:** Generation time of lactic acid bacteria in MRS broth containing different *Artemisia herba-alba* extracts (h; mean ± standard deviation).

Lactic Acid Bacteria Strain	MRS Broth	MRS Broth + *A. herba-alba* Ethanol Extract (5 mg/mL)	MRS Broth + *A. herba-alba* Hot-Water Extract (25 mg/mL)
*Pediococcus pentosaceus* SMFM2016-GK	12.9 ± 0.6 ^Ac^*	13.5 ± 0.4 ^Ab^	13.3 ± 0.5 ^Ab^
*P. pentosaceus* SMFM2016-NK1	13.3 ± 0.4 ^Aab^	13.6 ± 0.8 ^Ab^	13.9 ± 0.2 ^Ab^
*P. pentosaceus* SMFM2016-NK2	13.2 ± 0.3 ^Aab^	13.3 ± 0.9 ^Ab^	13.3 ± 0.7 ^Ab^
*P. pentosaceus* SMFM2016-NK3	13.2 ± 0.4 ^Aab^	13.5 ± 0.6 ^Ab^	13.7 ± 0.6 ^Ab^
*P. pentosaceus* SMFM2016-SK	13.1 ± 0.4 ^Aab^	13.2 ± 0.9 ^Ab^	13.3 ± 0.5 ^Ab^
*Lactiplantibacillus plantarum* SMFM2016-RK	14.3 ± 0.3 ^Ab^	14.5 ± 0.6 ^Ab^	14.2 ± 0.5 ^Ab^
*P. pentosaceus* SMFM2016-NK4	13.0 ± 0.5 ^Aab^	13.4 ± 0.4 ^Ab^	13.2 ± 0.6 ^Ab^
*P. pentosaceus* SMFM2016-RK	13.1 ± 0.5 ^Aab^	13.9 ± 0.5 ^Ab^	13.8 ± 0.7 ^Ab^
*Lactilactobacillus curvatus* SMFM2016-NK	13.8 ± 0.5 ^Aab^	3.9 ± 0.4 ^Ab^	14.3 ± 0.5 ^Ab^
*Lacticaseibacillus rhamnosus* GG	16.7 ± 1.6 ^Ba^	25.6 ± 6.3 ^Aa^	15.7 ± 1.4 ^Ba^

* Means with the same row with different superscript capital letters and same column with different superscript small letters are significantly different (*p* < 0.05).

**Table 4:** Sizes of inhibition zones produced by bioconverted broth with lactic acid bacteria (mm; mean ± standard deviation) against *Aggregatibacter actinomycetemcomitans* ATCC43718 and *Fusobacterium nucleatum* ATCC10953.

Lactic Acid Bacteria Strain	MRS Broth		MRS Broth + *A. herba-alba* Ethanol Extract (5 mg/mL)		MRS Broth + *A. herba-alba* Hot-Water Extract (25 mg/mL)	
	*Aggregatibacter actinomycetemcomitans* ATCC43718	*Fusobacterium nucleatum* ATCC10953	*A. actinomycetemcomitans* ATCC43718	*F. nucleatum* ATCC10953	*A. actinomycetemcomitans* ATCC43718	*F. nucleatum* ATCC10953
*Pediococcus pentosaceus* SMFM2016-GK	1.0 ± 0.0 ^Ab^	1.5 ± 0.6 ^ABbc^	0.5 ± 0.6 ^Bd^	1.8 ± 0.3 ^Ac^	1.1 ± 0.3 ^Ab^	0.7 ± 0.6 ^Bc^
*P. pentosaceus* SMFM2016-NK1	1.0 ± 0.0 ^Ab^	1.3 ± 0.5 ^Abc^	1.0 ± 0.0 ^Ac^	1.4 ± 0.5 ^Ac^	1.1 ± 0.3 ^Ab^	0.3 ± 0.5 ^Bc^
*P. pentosaceus* SMFM2016-NK2	1.3 ± 0.3 ^Ab^	1.4 ± 0.5 ^ABbc^	1.0 ± 0.0 ^Ac^	1.8 ± 0.3 ^Ac^	1.1 ± 0.3 ^Ab^	0.7 ± 0.6 ^Bc^
*P. pentosaceus* SMFM2016-NK3	1.0 ± 0.0 ^Ab^	1.5 ± 0.9 ^Abc^	1.0 ± 0.0 ^Ac^	1.2 ± 1.0 ^Ac^	1.1 ± 0.3 ^Ab^	0.0 ± 0.0 ^Bc^
*P. pentosaceus* SMFM2016-SK	1.0 ± 0.0 ^Ab^	2.0 ± 0.5 ^Ab^	1.0 ± 0.0 ^Ac^	1.6 ± 0.5 ^Ac^	0.8 ± 0.8 ^Ab^	0.3 ± 0.5 ^Bc^
*Lactiplantibacillus plantarum* SMFM2016-RK	1.4 ± 0.5 ^Ca^	3.1 ± 0.3 ^Aa^	2.1 ± 0.3 ^Aa^	2.8 ± 0.9 ^Ab^	2.0 ± 0.0 ^Ba^	3.1 ± 0.3 ^Aa^
*P. pentosaceus* SMFM2016-NK4	1.0 ± 0.4 ^Ab^	0.9 ± 0.6 ^Ac^	1.0 ± 0.0 ^Ac^	1.1 ± 0.3 ^Ac^	0.9 ± 0.3 ^Ab^	0.8 ± 0.3 ^Ac^
*P. pentosaceus* SMFM2016-RK	1.0 ± 0.0 ^Ab^	0.8 ± 0.5 ^Ac^	1.1 ± 0.3 ^Abc^	1.0 ± 0.0 ^Ac^	1.0 ± 0.0 ^Ab^	1.0 ± 0.0 ^Abc^
*Lactilactobacillus curvatus* SMFM2016-NK	1.0 ± 0.0 ^Bb^	3.1 ± 1.0 ^Ba^	1.9 ± 0.3 ^Aab^	4.0 ± 0.9 ^Aa^	1.6 ± 0.5 ^Aa^	2.6 ± 0.9 ^Ba^
*Lacticaseibacillus rhamnosus* GG	1.0 ± 0.0 ^Bb^	1.5 ± 0.6 ^Abc^*	1.5 ± 0.6 ^Ab^	2.1 ± 0.9 ^Abc^	1.8 ± 0.5 ^Aa^	1.8 ± 0.5 ^Ab^

* Means with the same row with different superscript capital letters and same column with different superscript small letters are significantly different (*p* < 0.05).

**Table 5:** α-glucosidase inhibitory activities (%; mean ± standard deviation) of bioconverted broths with lactic acid bacteria.

Lactic Acid Bacteria Strain	MRS Broth	MRS Broth+ *A. herba-alba* Ethanol Extract (5 mg/mL)	MRS Broth+ *A. herba-alba* Hot-Water Extract (25 mg/mL)
Positive control (*Lacticaseibacillus rhamnosus* GG)	83.2 ± 1.7 ^Aa^*	60.0 ± 5.5 ^Ba^	26.2 ± 0.9 ^Cb^
*Lactiplantibacillus plantarum* SMFM2016-RK	85.2 ± 0.3 ^Aa^	60.8 ± 5.0 ^Ba^	35.0 ± 0.2 ^Ca^
*Lactilactobacillus curvatus* SMFM2016-NK	0.0 ± 0.0 ^Bb^	2.7 ± 1.2 ^Ab^	5.9 ± 2.6 ^Ac^

* Means with the same row with different superscript capital letters and same column with different superscript small letters are significantly different (*p* < 0.05).

**Table 6:** pH and lactic acid bacteria cell count (mean ± standard deviation) of the bioconverted milk with *Lactiplantibacillus plantarum* SMFM2016-RK and *Artemisia herba-alba* extracts during fermentation at 35 °C.

Sample	0 h		6 h		24 h		37 h	
	pH	Cell Counts (Log CFU/mL)	pH	Cell Counts (Log CFU/mL)	pH	Cell Counts (Log CFU/mL)	pH	Cell Counts (Log CFU/mL)
BM1	6.57 ± 0.08	8.3 ± 0.0	-	-	4.42 ± 0.57	8.8 ± 0.2	-	-
BM2	6.39 ± 0.07	8.3 ± 0.0	-	-	-	-	4.58 ± 0.50	8.8 ± 0.3
BM3	6.20 ± 0.05	8.3 ± 0.2	4.62 ± 0.30	9.2 ± 0.1	-	-	-	-
BM4	6.11 ± 0.05	8.2 ± 0.1	4.58 ± 0.25	9.2 ± 0.1	-	-	-	-

BM1: bioconverted milk with *L. plantarum* SMFM2016-RK, BM2: BM1 and 5 mg/mL *A. herba-alba* ethanol extract, BM3: BM1 and 25 mg/mL *A. herba-alba* hot-water extract, BM4: BM1, 5 mg/mL *A. herba-alba* ethanol extract, and 25 mg/mL *A. herba-alba* hot-water extract.

**Table 7:** Sizes (mm; mean ± standard deviation) of inhibition zones produced by bioconverted milk with *Lactiplantibacillus plantarum* SMFM2016-RK and *Artemisia herba-alba* extracts against periodontal pathogens.

Sample	Periodontal Pathogen			Total Average
	*Aggregatibacter actinomycetemcomitans* ATCC43718	*Fusobacterium nucleatum* ATCC10953	*Porphyromonas gingivalis* ATCC33277	
10% skim milk	0.0 ± 0.0 ^D^	0.0 ± 0.0 ^D^	0.0 ± 0.0 ^D^	0.0 ± 0.0
BM1	2.6 ± 1.1 ^B^	1.6 ± 0.5 ^C^	1.9 ± 0.3 ^BC^	2.0 ± 0.8
BM2	3.0 ± 0.8 ^AB^	2.5 ± 0.6 ^BC^	1.8 ± 0.5 ^BC^	2.4 ± 0.8
BM3	3.6 ± 0.8 ^A^	1.9 ± 1.0 ^BC^	1.9 ± 0.3 ^BC^	2.5 ± 1.1
BM4	3.3 ± 1.5 ^AB^	2.9 ± 0.6 ^AB^	1.6 ± 0.5 ^C^	2.6 ± 1.1

BM1: bioconverted milk with *L. plantarum* SMFM2016-RK, BM2: BM1 and 5 mg/mL *A. herba-alba* ethanol extract, BM3: BM1 and 25 mg/mL *A. herba-alba* hot-water extract, BM4: BM1, 5 mg/mL *A. herba-alba* ethanol extract, and 25 mg/mL *A. herba-alba* hot-water extract; A–D; different letters indicate a significant difference (*p* < 0.05).

**Table 8:** Gene ontology by gene prediction of *Lactiplantibacillus plantarum* SMFM2016-RK chromosome.

Category	Gene Ontology	Number of Transcripts
Cellular component	Cell part	263
	Cell	263
	Protein-containing complex	120
	Organelle	61
	Extracellular region	5
	Membrane	397
	Membrane part	249
	Extracellular region part	4
	Organelle part	16
Molecular function	Catalytic activity	1087
	Binding	805
	Molecular carrier activity	2
	Transport activity	219
	Antioxidant activity	8
	Transcription regulator activity	105
	Molecular function regulator	1
	Structural molecule activity	51
	Molecular transducer activity	2
Biological process	Localization	317
	Response to stimulus	104
	Metabolic process	1089
	Cellular process	845
	Biological regulation	223
	Regulation of biological process	217
	Cellular component organization or biogenesis	56
	Negative regulation of biological process	9
	Multi-organism process	9
	Signaling	22
	Developmental process	9
	Immune system process	1
	Biological adhesion	8
	Detoxification	2
	Carbon utilization	1

**Table 9:** Distribution of predicted open reading frames (ORFs) over functional class in *Lactiplantibacillus plantarum* SMFM2016-RK.

Description	Number of ORFs	Ratio (%)
Translation, ribosomal structure, and biogenesis	150	4.7847
Transcription	258	8.2297
Replication, recombination, and repair	196	6.2520
Cell cycle control, cell division, chromosome partitioning	26	0.8293
Defense mechanisms	66	2.1053
Signal transduction mechanisms	70	2.2329
Cell wall/membrane/envelope biogenesis	179	5.7097
Cell motility	4	0.1276
Intracellular trafficking, secretion, and vesicular transport	25	0.7974
Posttranslational modification, protein turnover, chaperones	69	2.2010
Energy production and conversion	111	3.5407
Carbohydrate transport and metabolism	290	9.2504
Amino acid transport and metabolism	206	6.5710
Nucleotide transport and metabolism	86	2.7432
Coenzyme transport and metabolism	63	2.0096
Lipid transport and metabolism	62	1.9777
Inorganic ion transport and metabolism	125	3.9872
Secondary metabolites biosynthesis, transport, and catabolism	20	0.6380
General function prediction only	349	11.1324
Function unknown	780	24.8804
Total	3135	100

**Table 10:** Coding DNA sequences (DNA) related to proteolysis and amino acid metabolism identified in the gene annotation of *Lactiplantibacillus plantarum* SMFM2016-RK.

Start	End	Product	Gene	Identity	e-Value	Bit Score
1,849,164	1,850,129	Oligopeptide transport ATP-binding protein OppF	*oppF*	77.636	0.0	518
1,850,136	1,851,215	Oligopeptide transport ATP-binding protein OppD	*oppD*	79.883	0.0	570
2,470,957	2,472,429	Di-/tripeptide transporter	*dtpT*	66.189	0.0	648
962,965	964,776	Oligoendopeptidase F, plasmid	*pepF1*	57.333	0.0	728
3,234,367	3,236,283	Neutral endopeptidase	*pepO*	59.528	0.0	791
50,4486	505,796	D-serine dehydratase	*dsdA*	57.619	9.40 × 10^−175^	501
2,530,776	2,531,678	L-serine dehydratase, alpha chain	*sdhA*	72.069	5.73 × 10^−138^	397
820,964	822,202	Serine hydroxymethyltransferase	*glyA*	70.270	0.0	607
1,173,945	1,175,741	Aspartate--tRNA ligase	*aspS*	74.617	0.0	917
2,291,674	2,292,909	Argininosuccinate synthase	*argG*	73.350	0.0	634
2,290,271	2,291,674	Argininosuccinate lyase	*argH*	71.024	0.0	699

**Table 11:** Coding DNA sequences (DNA) related to phenolic acid decarboxylase identified in the gene annotation of *Lactiplantibacillus plantarum* SMFM2016-RK.

Start	End	Product	Gene	Identity	e-Value	Bit Score
335,672	337,144	Gallate decarboxylase	*lpdC*	82.857	0	841
3,008,485	3,009,021	Phenolic acid decarboxylase PadC	*padC*	87.64	3.70 × 10^−116^	332

## Data Availability

The original contributions presented in the study are included in the article, further inquiries can be directed to the corresponding authors.

## References

[1] WHO (World Health Organization) WHO Highlights Oral Health Neglect Affecting Nearly Half of the World’s Population. https://www.who.int/news/item/18-11-2022-who-highlights-oral-health-neglect-affecting-nearly-half-of-the-world-s-population.

[2] Borgnakke W.S. (2019). IDF Diabetes Atlas: Diabetes and oral health—A two-way relationship of clinical importance. Diabetes Res. Clin. Pract..

[3] Ahmad R., Haque M. (2021). Oral health messiers: Diabetes mellitus relevance. Diabetes Metab. Syndr. Obes..

[4] Van de Laar F.A., Lucassen P.L., Akkermans R.P., van de Lisdonk E.H., Rutten G.E., van Weel C. (2005). α-Glucosidase inhibitors for patients with type 2 diabetes: Results from a Cochrane systematic review and meta-analysis. Diabetes Care.

[5] Basri R., Ullah S., Halim S.A., Alharthy R.D., Rauf U., Khan A., Hussain J., Al-Ghafri A., Al-Harrasi A., Shafiq Z. (2023). Synthesis, biological evaluation, and molecular docking study of chromen-linked hydrazine carbothioamides as potent α-glucosidase inhibitors. Drug Dev. Res..

[6] Forozan R., Ghomi M.K., Iraji A., Montazer M.N., Noori M., Dastyafteh N., Mojtabavi S., Faramarzi M.A., Sadat-Ebrahimi S.E., Larijani B. (2023). Synthesis, in vitro inhibitor screening, structure–activity relationship, and molecular dynamic simulation studies of novel thioquinoline derivatives as potent α-glucosidase inhibitors. Sci. Rep..

[7] Ullah S., Waqas M., Halim S.A., Khan I., Khalid A., Abdalla A.N., Makeen H.A., Ibrar A., Khan A., Al-Harrasi A. (2023). Triazolothiadiazoles and triazolothiadiazines as potent α-glucosidase inhibitors: Mechanistic insights from kinetics studies, molecular docking and dynamics simulations. Int. J. Biol. Macromol..

[8] Huligere S.S., Chandana Kumari V.B., Alqadi T., Kumar S., Cull C.A., Amachawadi R.G., Ramu R. (2023). Isolation and characterization of lactic acid bacteria with potential probiotic activity and further investigation of their activity by α-amylase and α-glucosidase inhibitions of fermented batters. Front. Microbiol..

[9] Haque M.M., Yerex K., Kelekis-Cholakis A., Duan K. (2022). Advances in novel therapeutic approaches for periodontal diseases. BMC Oral Health.

[10] Rosas-Val P., Adhami M., Brotons-Canto A., Gamazo C., Irache J.M., Larrañeta E. (2023). 3D printing of microencapsulated *Lactobacillus rhamnosus* for oral delivery. Int. J. Pharm..

[11] Mahasneh S.A., Mahasneh A.M. (2017). Probiotics: A promising role in dental health. Dent. J..

[12] Rejiniemon T.S., Hussain R.R., Rajamani B. (2015). *In-vitro* functional properties of *Lactobacillus plantarum* isolated from fermented ragi malt. South Indian J. Biol. Sci..

[13] Iwasaki K., Maeda K., Hidaka K., Nemoto K., Hirose Y., Deguchi S. (2016). Daily intake of heat-killed *Lactobacillus plantarum* L-137 decreases the probing depth in patients undergoing supportive periodontal therapy. Oral Health Prev. Dent..

[14] Pudgar P., Povšič K., Čuk K., Seme K., Petelin M., Gašperšič R. (2021). Probiotic strains of *Lactobacillus brevis* and *Lactobacillus plantarum* as adjunct to non-surgical periodontal therapy: 3-month results of a randomized controlled clinical trial. Clin. Oral Investig..

[15] Lin C.W., Chen Y.T., Ho H.H., Hsieh P.S., Kuo Y.W., Lin J.H., Liu C.R., Huang Y.F., Chen C.W., Hsu C.H. (2022). Lozenges with probiotic strains enhance oral immune response and health. Oral Dis..

[16] Dadgar S., Heydarian A., Sobouti F., Goli H., Rakhshan V., Heidari M. (2021). Effects of probiotic and fluoride mouthrinses on *Streptococcus mutans* in dental plaque around orthodontic brackets: A preliminary explorative randomized placebo-controlled clinical trial. Dent. Res. J. (Isfahan).

[17] Zhu Y., Chen J., Ji X., Hu X., Ling T., Zhang Z., Bao G., Wan X. (2015). Changes of major tea polyphenols and production of four new B-ring fission metabolites of catechins from post-fermented Jing-Wei Fu brick tea. Food Chem..

[18] Luo J., Liu S., Lu H., Chen Q., Shi Y. (2023). Microbial Community Variations and Bioconversion Improvements during Soybean-Based Fermentation by Kefir Grains. Foods.

[19] Cosier D., Lambert K., Batterham M., Sanderson-Smith M., Mansfield K.J., Charlton K. (2024). The INHABIT (synergIstic effect of aNtHocyAnin and proBIoTics in) Inflammatory Bowel Disease trial: A study protocol for a double-blind, randomised, controlled, multi-arm trial. J. Nutr. Sci..

[20] Moufid A., Eddouks M. (2012). *Artemisia herba alba*: A popular plant with potential medicinal properties. Pak. J. Biol. Sci..

[21] Jang H.J. (2018). Potential Use of Lactic Acid Bacteria Isolated from Kimchi as Probiotics. Master’s Thesis.

[22] Choi Y., Park E., Kim S., Ha J., Oh H., Kim Y., Lee Y., Seo Y., Kang J., Lee S. (2021). Alleviation of periodontal disease using *Lactobacillus curvatus* SMFM2016-NK. J. Funct. Foods.

[23] Hunt R. (1982). Plant growth curves. The Functional Approach to Plant Growth Analysis.

[24] Souhila T., Fatma Zohra B., Tahar H.S. (2019). Identification and quantification of phenolic compounds of *Artemisia herba-alba* at three harvest time by HPLC–ESI–Q-TOF–MS. Int. J. Food Prop..

[25] Mohammed M.J., Anand U., Altemimi A.B., Tripathi V., Guo Y., Pratap-Singh A. (2021). Phenolic composition, antioxidant capacity and antibacterial activity of white wormwood (*Artemisia herba-alba*). Plants.

[26] Gallego R., Montero L., Cifuentes A., Ibáñez E., Herrero M. (2018). Green extraction of bioactive compounds from microalgae. J. Anal. Test..

[27] Mangia N.P., Saliba L., Deiana P. (2019). Functional and safety characterization of autochthonous Lactobacillus paracasei FS103 isolated from sheep cheese and its survival in sheep and cow fermented milks during cold storage. Ann. Microbiol..

[28] Kim H., Kim J., Kim Y., Jeong Y., Kim J., Paek N., Kang C. (2020). Antioxidant and probiotic properties of Lactobacilli and Bifidobacteria of human origins. Biotechnol. Bioprocess Eng..

[29] Nanno M., Morotomi H., Takayama H., Kuroshima T., Tanaka R., Mutai M. (1986). Mutagenic activation of biliary metabolites of benzo (a) pyrene by β-glucuronidase-positive bacteria in human faeces. J. Med. Microbiol..

[30] Rafter J. (2002). Lactic acid bacteria and cancer: Mechanistic perspective. Br. J. Nutr..

[31] Monteagudo-Mera A., Caro I., Rodríguez-Aparicio L.B., Rúa J., Ferrero M.A., García-Armesto M.R. (2011). Characterization of certain bacterial strains for potential use as starter or probiotic cultures in dairy products. J. Food Prot..

[32] Rice-Evans C., Miller N., Paganga G. (1997). Antioxidant properties of phenolic compounds. Trends Plant Sci..

[33] Ebringer L., Ferenčík M., Krajčovič J. (2008). Beneficial health effects of milk and fermented dairy products. Folia Microbiol. (Praha).

[34] Ostaff M.J., Stange E.F., Wehkamp J. (2013). Antimicrobial peptides and gut microbiota in homeostasis and pathology. EMBO Mol. Med..

[35] Oboh G., Ogunsuyi O.B., Ogunbadejo M.D., Adefegha S.A. (2016). Influence of gallic acid on α-amylase and α-glucosidase inhibitory properties of acarbose. J. Food Drug Anal..

[36] Ramchandran L., Shah N.P. (2008). Proteolytic profiles and angiotensin-I converting enzyme and α-glucosidase inhibitory activities of selected lactic acid bacteria. J. Food Sci..

[37] Kwun S.Y., Bae Y.W., Yoon J.A., Park E.H., Kim M.D. (2020). Isolation of acid tolerant lactic acid bacteria and evaluation of α-glucosidase inhibitory activity. Food Sci. Biotechnol..

[38] Dar M.A., Siddiqui N.A., Mir S.R., Akbar S., Mothana R.A., Masoodi M.H. (2024). Anti-diabetic activity-guided isolation of α-amylase and α-glucosidase inhibitory terpenes from *Capsella bursa-pastoris* Linn. Open Chem..

[39] Younsi F., Trimech R., Boulila A., Ezzine O., Dhahri S., Boussaid M., Messaoud C. (2016). Essential oil and phenolic compounds of Artemisia herba-alba (Asso.): Composition, antioxidant, antiacetylcholinesterase, and antibacterial activities. Int. J. Food Prop..

[40] Loo Y.T., Howell K., Chan M., Zhang P., Ng K. (2020). Modulation of the human gut microbiota by phenolics and phenolic fiber-rich foods. Compr. Rev. Food Sci. Food Saf..

[41] Leonard W., Zhang P., Ying D., Fang Z. (2021). Hydroxycinnamic acids on gut microbiota and health. Compr. Rev. Food Sci. Food Saf..

[42] Sun L., Miao M. (2020). Dietary polyphenols modulate starch digestion and glycaemic level: A review. Crit. Rev. Food Sci. Nutr..

[43] Varghese N.J., Mukherjee S., Ivanova N., Konstantinidis K.T., Mavrommatis K., Kyrpides N.C., Pati A. (2015). Microbial species delineation using whole genome sequences. Nucleic Acids Res..

